# Disrupted Sarcomere Reorganization of Cardiomyopathy-Prone Human iPSC-Derived Cardiomyocytes on a Dynamic Mechanical Substrate

**DOI:** 10.1007/s12195-025-00880-z

**Published:** 2025-11-21

**Authors:** Nhu Y. Mai, Xiangjun Wu, Huiyao Liu, Ariel Ash-Shakoor, Huaiyu Shi, Zhuocheng Qu, Patrick T. Mather, Xinrui Wang, James H. Henderson, Zhen Ma

**Affiliations:** 1 Department of Biomedical and Chemical Engineering, Syracuse University, 318 Bowne Hall, Syracuse, NY 13244 USA; 2 BioInspired Institute for Materials and Living Systems, Syracuse University, Syracuse, NY USA; 3 Department of Pharmacology, SUNY Upstate Medical University, Syracuse, NY USA; 4 Department of Chemical Engineering, Penn State University, University Park, PA USA

**Keywords:** Active biomaterials, Dynamic mechanobiology, Human induced pluripotent stem cells, Disease modeling

## Abstract

**Introduction:**

Approximately 15% of dilated cardiomyopathy (DCM) cases are associated with Bcl2-associated athanogene 3 (BAG3) gene mutations, which play a crucial role in myofilament organization and contractile behavior. Previous studies have highlighted the role of dynamic mechanical stress in myofibril alignment in human-induced pluripotent stem cell-derived cardiomyocytes (hiPSCCMs). In this study, we employed thermo-responsive shape memory polymers (SMPs) to mimic the dynamic mechanical environment of the extracellular matrix (ECM) and investigated their impact on myofibril assembly in healthy wild-type (WT) and BAG3 knockout (BAG3-/-) hiPSC-CMs

**Methods:**

We synthesized Tert-Butyl Acrylate (TBA) and Butyl acrylate (BA)-based SMP substrate. hiPSC-CMs were cultured on 30oC on 40% strained (dynamic) and un-strain (static) SMPs for two days before proceeding with polymer recovery at 37oC. Myofibril components of BAG3 knock-out (KO) and WT CMs were evaluated by immunocytochemistry fluorescent images at 5 hours and 24 hours after triggering the shape changes of the SMP substrate. We quantified Z-lines and M-lines of hiPSC-CMs to evaluate sarcomere remodeling on static and dynamic substrates.

**Results:**

Our findings revealed that BAG3-/- hiPSC-CMs exhibited persistent Z-line disruption in sarcomeres compared with WT hiPSC-CMs, but M-line structures were less sensitive to mechanical stress at 5 hours, highlighted a temporal distinction in the assembly and regulation of Z-lines over M-lines. While no significant changes were detected at 5 hours, BAG3-/- CMs exhibited similar impairments in M-line organization as seen with Z-lines. These findings suggest that M-lines in BAG3-/- CMs display heightened sensitivity to dynamic mechanical actuation, but this phenotype emerges only after prolonged culture.

**Conclusions:**

This study highlights the interplay between genetic deficiency and mechanical stress to facilitate disease progression in BAG3-associated DCM.

**Supplementary Information:**

The online version contains supplementary material available at 10.1007/s12195-025-00880-z.

## Introduction

Sarcomeres are fundamental contractile units of myofibrils in cardiomyocytes. Actin and myosin are the primary components of thin and thick filaments anchored to Z-lines that support the integrity of sarcomeres [1]. Titin, which connects thick filaments to the Z-lines, provides elasticity through the M-bands composed of myomesin. Numerous mutations in sarcomere genes have been identified as related to cardiomyopathy, including dilated cardiomyopathy (DCM). These sarcomere mutations disrupt myofibril formation, induce sarcomere disarray, and impair the contractile functions of cardiomyocytes, leading to various cardiomyopathies [2, 3]. In DCM cardiomyocytes, Z-line protein localization is compromised, with proteins found in the nuclear region instead of in sarcomeres [4].

Bcl2-associated athanogene 3 (BAG3) gene mutations have been found to be associated with approximately 15% of DCM cases [5]. BAG3 plays a central role in protein quality control through the autophagic clearance of misfolded proteins such as myosins, desmin, and titin, thus maintaining protein homeostasis and structural integrity in cardiomyocytes [6]. BAG3 mutations have been widely reported to cause myofibrillar disorganization and reduced contractile function in mice [7, 8]. Neonatal cardiomyocytes from BAG3 knockout mice showed rapid disruption of the myofibril structure under mechanical stretch, indicating the critical role of the BAG3 protein in dynamic sarcomere remodeling [9]. Human iPSC-derived cardiomyocytes (hiPSC-CMs) also show that BAG3 mutations disrupt myofibril structure, impact chaperone networks, and compromise contractile functions upon exposure to proteasome inhibitors [10, 11]. More recently, BAG3 was found to engage with mechanotransduction signaling in myoblasts in response to dynamic changes in the mechanical environment [12, 13]. These studies suggest that the BAG3-dependent protein quality control network in cardiomyocytes is essential for maintaining myofibrillar structures in a dynamically changing mechanical environment.

To gain deeper insight into the mechanobiological aspects of cardiac diseases, various in vitro cardiac models have been developed to explore how different mechanical inputs affect heart tissue physiology [14–16]. Given the dynamic nature of the mechanical microenvironment experienced by cardiomyocytes in vivo, there is growing emphasis on developing model systems capable of providing dynamic mechanical inputs during disease progression [17, 18]. For example, a recent study used a magnetorheological elastomer substrate to dynamically control the cardiac afterload of 3D microheart muscles [19]. Shape memory polymers (SMPs) are a class of materials capable of “memorizing” and returning to their original shapes when triggered by specific stimuli. They have been used to fabricate topography-programmed dynamic substrates that can influence cell alignment, migration, and cytoskeletal organization [20–22]. Particularly in cardiomyocytes, sarcomere alignment is highly affected by the extracellular mechanical environment. In our previous work, we achieved on-demand topographic transition on an SMP substrate to mimic dynamic changes in the extracellular microenvironment of a developing myocardium [21]. We found that SMP can effectively promote hiPSC-CM elongation and induce progressive myofibril reorganization and growth [21, 23]. Although SMPs are being widely developed for studying cell mechanobiology in dynamic mechanical environments, to the best of our knowledge, the use of SMPs for modeling human diseases remains largely untapped.

To our knowledge, this is the first study to report the use of an SMP-based dynamic substrate to study myofibril disease phenotypes associated with genetic DCM. Our SMPs can fully recover their shape within 4 h at 37 °C in hydrated environments, while retaining their shapes at room temperature under dry conditions [24]. This property of memory enables us to investigate the synergy between genetic deficiency and mechanical stress in myofibril diseases. Wild-type (WT) and BAG3-/- iPSC-CMs were used to investigate myofibril remodeling on dynamic SMP substrates. Sarcomere Z-lines and M-lines from BAG3-/- hiPSC-CMs were examined to elucidate myofibril remodeling. We found that the BAG3 mutation impaired the myofibril reorganization of hiPSC-CMs under mechanical stress. We highlighted that the dynamic mechanical environment facilitated the manifestation of BAG3-associated myofibril disarray in hiPSC-CMs compared with a static environment.

## Materials and Methods

### SMP Fabrication

The SMPs were cross-linked with tert-butyl acrylate (tBA) (Acros Organics, Ca# 371130010) and butyl acrylate (BA) ((Sigma-Aldrich, Ca# 234923). Triethylene glycol dimethacrylate (TEG-DMA) (Sigma-Aldrich, Ca# 86680) and 2,2-dimethoxy-2-phenylacetophenone (DMPA) ((Sigma-Aldrich, Ca# 196118) were used as crosslinkers and photoinitiators, respectively. The monomer solution was prepared from tBA:BA in a ratio of 95:5 by weight, 1 wt% DMPA, and 5 wt% TEGDMA of the total solution weight. The solution was injected into two Rain-X-coated glass slides sandwiched by a 1 mm thick Teflon spacer and then crosslinked under UV light (Spectronics XL-1000 UV crosslinker) for 1 h. The unreacted monomers were removed from the crosslinked polymer by immersion in 50% methanol overnight. The polymers were air-dried in a vacuum oven at 40 °C for 24 h to remove water in the matrix after air-drying at room temperature for one day. The synthesized polymers were cut into rectangular shapes with a length of 23 mm and width of 5 mm. The polymers were pre-heated at 70 °C for 15 min and stretched at 40% strain (140% of the original length). Stretching can be programmed by manual stretching or through use of a Dynamic Mechanical Analyzer (DMA) in controlled quasi-static mode. The stretched polymers were then cooled to room temperature before unloading. To prevent shape recovery, stretched polymers were stored at 4 °C.

### Differential Scanning Calorimeter (DSC)

DSC Q200 was used to measure the glass transition temperature (Tg) of the SMPs. samples (*n* = 3, weighing 3–5 mg) were placed in T-zero aluminum pans and equilibrated at − 40 °C. The samples were heated to 150 °C at a rate of 10 °C/min, held isothermally for 2 min, cooled to 50 °C at 5 °C/min, and then held isothermally for 20 min. Next, the samples were cooled to − 40 °C, held isothermally for 2 min, and finally reheated to 150 °C at 10 °C/min. *Tg* was analyzed in the second cycle. For the wet conditions, samples were immersed in deionized (DI) water at 60 °C for 20 min before being transferred to hermetic pans. These samples underwent a single heating cycle at 150 °C at a rate of 10 °C/min, and *Tg* was determined from the resulting thermal curve.

### Shape Memory Characterization

Polymers (*n* = 3) were cut into dog-bone shapes with a gauge length of 6.25 mm and width of 1.5 mm (1/4-scale ASTM D639 Type IV). TA Instruments DMA Q800 was set up in controlled-force mode to examine the shape-memory profile. The specimens were anchored to clamps inside the thermal chamber. The polymer samples were subjected to three deformation and recovery cycles. The polymers were heated to 60 °C and then stretched to 40% strain with a controlled force of 0.03 N/min. Nitrogen gas was applied to cool polymers to − 5 °C at 2 °C/min to enable shape fixing in the elongated state, followed by stress removal. Polymers were next heated to 60 °C under a minimal tensile load of 1 mN as the strain was recovered under the action of the network’s rubber elasticity. This cycle was repeated two more times. For each, shape memory cycle, the fixing ratio and recovery capacity were measured by Eqs. 1 and 2, respectively, in which ${\epsilon }_{u}$ is the strain after shape fixed, ${\epsilon }_{m}(L)$ is the maximum strain during deforming, ${\epsilon }_{p}$ is the permanent strain, and *N* is the cycle number of shape memory testing.

1 $${R}_{f}=\frac{{\epsilon }_{u}}{{\epsilon }_{m}(L)}$$

2 $${R}_{r}=\frac{{\epsilon }_{m}-{\epsilon }_{p}(N)}{{\epsilon }_{m}-{\epsilon }_{p}(N-1)}$$

### Shape Recovery in Wet Condition

The polymers were cut into small pieces (*n* = 12) with a width of 4 mm and incubated in DPBS at 37 °C for 5 h. The shape changes were recorded hourly (Fig. 2b). The recovery percentage was calculated using Eq. (3), where l_0_ is the initial pattern width and *l*_*r*_ is the pattern width after recovery.

3 $$Recover \:percentage (\%)=\frac{{l}_{0}-{l}_{r}}{{l}_{0}}\times 100$$

### hiPSCs Culture

hiPSCs (WTC and BAG3-/-) were obtained from Dr. Conklin’s laboratory at the Gladstone Institute of Cardiovascular Research [11]. hiPSCs were cultured in 6-well plate (Corning) in Essential 8 (E8) medium (Thermo Fisher Scientific). Culture surfaces were coated with Geltrex (Ca# A1413302; Thermo Fisher Scientific). iPSCs were passaged when the confluency reached 90% with a density of 2.5 × 10^4^ cells/cm^2^. For the first 24 h, the medium was supplemented with 10 μM of ROCK inhibitor (Y-27632; Stem Cell Technology, Ca# 72308). E8 medium was refreshed daily.

### hiPSC-CM Differentiation and Purification

Cardiomyocyte differentiation was performed with 9 uM GSK3 inhibitor (CHIR99021) in RPMI supplemented with B27 minus insulin (B27/-I) on day 0 to induce mesoderm formation for the first 24 h. Cells were recovered in (B27/-I) for one day before being treated with 5uM WNT inhibitor (IWP4) on day 2 for 2 days. The cells were then cultured in RPMI supplemented with B27 complete medium (B27/+ C) from days 6 to 20. Purification was performed on day 20 to harvest the hiPSC-CMs. Differentiated hiPSC-CMs were dissociated using the STEMdiff CM dissociation kit following the manufacturer’s protocol. The cells were re-coated with Geltrex 6-well plates in B27/+ C media containing 10uM ROCK-i. Cells were recovered with B27/+ C media for two days, then treated with lactate medium. The lactate purification medium consisted of DMEM without glucose (Thermo Fisher Scientific), NEAA (Thermo Fisher Scientific), GlutaMAX (Thermo Fisher Scientific), and 4 mM lactate (Sigma-Aldrich). Purified CMs were cultured in B27/+ C media refreshed every 2 days.

### Cell Seeding on SMP

The polymers were cut into small pieces (3 mm long) and sanitized with ethanol and UV light for 1 h in a 48 well-plate. The sterile polymers were washed with PBS 3 times to remove the remaining ethanol. Geltrex was coated on the strain (dynamic) and unstrained (static) polymer substrates at room temperature for one day. The purified hiPSC-CMs were dissociated with 0.025% trypsin for 10 min, centrifuged at 800 rpm for 5 min, and resuspended in (B27/+ C). Single hiPSC-CMs were seeded at 3 × 10^4^ cells/well with a ROCK inhibitor. Cells were maintained in a 30 °C incubator with 5% CO_2_ for 48 h before switching to 37 °C to trigger shape memory recovery. Cells were able to attach and spread on the SMP surface. Cells were cultured under 37 °C for 5 h before immunostaining.

### Immunocytochemistry Staining

The samples were fixed in a 4% (vol/vol) paraformaldehyde (PFA) solution 5 h after recovery at 37 °C. A Triton solution of 0.2% was used to permeabilize the cell membranes for 5 min. CMs were blocked with 2% Bovine Serum Albumin (BSA) for 30 min. The cells were incubated at 37 °C with the primary and secondary antibodies (Table S1) at 2 and 1.5 h perceptively after washing with PBS three times. Nuclei were stained with DAPI solution for 10 min. Brightfield and fluorescent images were captured using a Nikon Eclipse Ti microscope equipped with a Zyla 4.2 PLUS sCMOS camera.

### Myofibril Remodeling Quantification

The hiPSC-CM fluorescent images were used to analyze cellular remodeling. The cell aspect ratio (AR), which is the ratio between length and width, represents cell elongation. The myofibrils were stained with α-actinin and myomesin. The myofibril characterization was performed using the zlineDetection algorithm in MATLAB available at https://github.com/Cardiovascular-Modeling-Laboratory/zlineDetection [25]. Z-line and M-line parameters (OOP, mean Z-line, M-line length, Z-line, and M-line distance) were calculated. Myofibril images (Fig. S1a) were converted into binary skeletons (Fig. S1b) to quantitate of Z-line orientational order. Z-lines are not always aligned perpendicular to the imaging plane; software creates the synthetic dataset, including overlaying color-coded segments (Fig. S1c) with variable orientations. Continuous line segments were measured in pixels, where distinct colors indicated different lengths. The orientational order parameter (OOP) was quantified as the average angle between two adjacent sarcomeres. The Z-line length was the length of the sarcomere Z-disc. The M-line length is the M-disc length. The Z-line and M-line distances were measured as the distance between two adjacent lines. Sarcomere density is determined by the number of sarcomeres counted from zlineDetection to the area of the individual cell. The cell area was measured by selecting a selective area from the ImageJ software.

### Contractile Behavior Analysis

Contractile activity of individual hiPSC-CMs was assessed using motion-tracking software, available at https://gladstone.org/46749d811 [26]. A Nikon Ti-E inverted microscope with Andor Zyla 4.2 + digital CMOS camera was used to capture beating video. Contracting CMs were recorded at 41 frames per second for 10 s in Brightfield (Movies S1–S4) and exported as a single-frame series, then were analyzed by a Motion Tracking algorithm. The motion vectors were documented based on the pixel movement and represented contractile physiology. Beating video of hiPSC-CM beating was analyzed by the software, to output beat rate (beats per minute), contraction-relaxation time interval (C-R interval), contraction velocity (pixel per second), relaxation velocity (pixel per second). The time interval was corrected based on the beat rate as follow.

$$Corrected \:Time \: Interval =\frac{time \:interval}{\sqrt{\frac{1}{beat \:rate (Hz)}}}$$

Each contractile measurement of BAG3 hiPSC-CMs was normalized to the corresponding average value from WT hiPSC-CMs.

### Statistical Analysis

To ensure robust and reproducible findings, we acquired at least three SMPs for biological replication, collected three to six images from each SMP substrate for technical replication, and analyze two to three cells from each image. Thus, we captured both inter-polymer variability and cell-to-cell heterogeneity in our measurements. Data was analyzed using the GraphPad Prism software. Data are presented as all data points with mean and standard deviation. Two-way ANOVA with multiple t-tests followed by Tukey’s multiple comparisons test to identify pairwise differences while controlling familywise error. Statistical significance was determined based on *p*-values < 0.05 (*), < 0.01 (**), < 0.001 (***), and < 0.0001 (****). The sample sizes for each condition at different time points are *n* ≥ 15 for sarcomere analysis and *n* ≥ 6 for function analysis.

## Results

### Dynamic Shape Changes of tBA-BA SMP

**Fig. 1:**
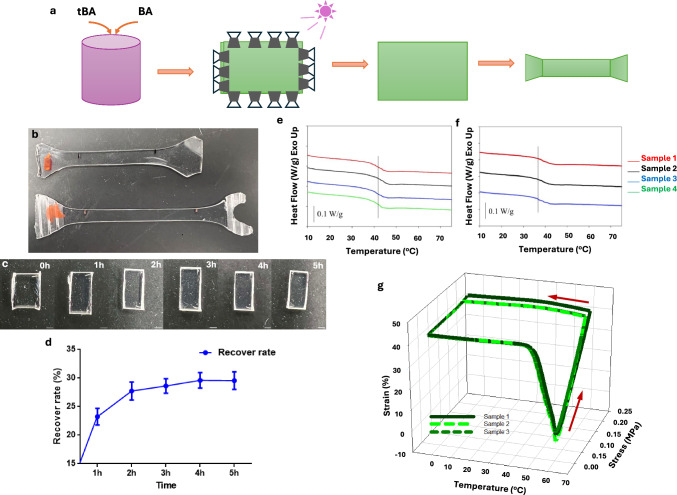
The SMP fabrication and mechanical characterization. **a** Schematic of the fabrication of tBA-BA co-polymer SMP substrate. **b** The SMP was manually stretched to 40% strain (Scale bar: 1 cm). **c** Recovery of the SMP at 37 °C in DPBS over a period of 5 hours is depicted (Scale bar: 1 mm). **d** The recovery rate of the SMP at 37 °C in DPBS after 5 hours is presented. The Tg of the SMPs were approximately 43 °C in **e** dry conditions and 37 °C in **f** wet conditions, as measured by differential scanning calorimetry (DSC). **g** A 3-D plot of the dynamic mechanical analysis (DMA) cycles revealed the SMP can deform and retain its shapes in high-temperature and low-temperature segments with applied stress, respectively

After curing under UV light, TBA-co-BA SMPs were cut into dog-bone shapes and pre-heated to 60 °C in an oven for manual stretching to 40% strain (Fig. 1a, b). The stretched polymers were cut into small pieces for cell culture. We triggered a shape-memory effect on our SMP by increasing the temperature. To profile the SMP recovery rate, the polymer width was measured hourly at 37 °C during water immersion for 5 h (Fig. 1c). The rapid shape change occurred in the first two hours and ceased at 5 h, returning to its original shape (Fig. 1d). DSC was used to determine the thermal transition temperatures under both the dry and wet conditions. The Tg value of the dry polymer was approximately 43 °C (Fig. 1e), and the polymer Tg under wet conditions was 38 °C (Fig. 1f). DSC testing was also conducted on the wet samples to better simulate the aqueous environment of the human body. The samples were immersed in water at 60 °C for 20 min, plasticization resulted a reduction in Tg. This plasticization effect increases chain mobility by disrupting intermolecular interactions and altering the physical structure of the polymer [27]**.** The DSC results from wet and dry samples revealed that all transition temperatures of the SMPs consistently remained above body temperature and were relatively high compared to previously developed tBA-co-BA SMP systems [28].

A DMA apparatus was used to investigate the shape memory properties of SMPs. The polymer reached 40% strain at a temperature of 60 °C during deformation, and it was fixed at 42.68% of strain when cooling down to 40 °C. The force applied to maintain the deformed shape of the polymer was removed at − 10 °C. A slight recovery was observed; however, the polymer retained its temporary shape. After the polymer was stimulated with heat, significant shape recovery was recorded at temperature of 45 °C, and the polymer was completely recovered at 60 °C. To examine the shape memory capacity, the heat-stretched polymer was cooled down to fix the strain in a vitrified state, followed by controlled heating that allowed the deformed polymer to return to the primary shape (Tr). The average fixing ratio (*R*_*f*_) and recovery ratio (*R*_*r*_) were used to evaluate the shape retention and regaining ability of the SMPs, respectively. The average fixing ratio (*R*_*f*_) was 94% and the recovery ratio (*R*_*r*_) was 95%, which indicated the capability to preserve the permanent shape from the deformation of the SMPs (Fig. 1g). Stimuli-responsive SMP substrates can provide dynamic mechanical cues for the study of cell mechanobiology [21, 29, 30].

### Myofibril Remodeling at 5 Hours After Shape Recovery Triggering

**Fig. 2:**
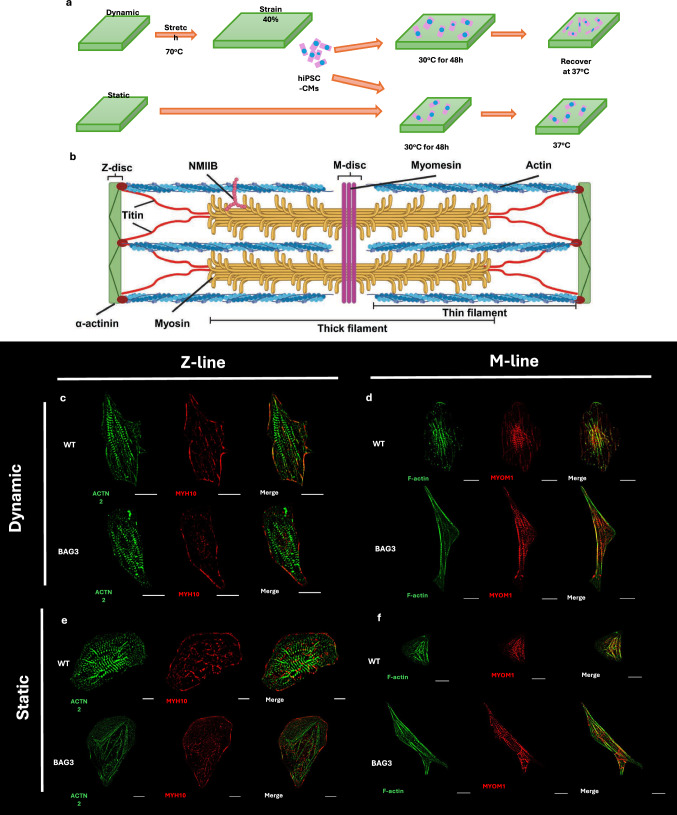
Sarcomere organization on both static and dynamic SMP substrate. **a** hiPSC-CMs were seeded onto static and dynamic SMP substrate with 40% strain. **b** Schematic illustration of cardiac myofibril structures. Fluorescent images of hiPSC-CMs on static and dynamic substrates 5 h after shape change triggered by the temperature stimuli, staining with ACTN2 and MYH10 on **c** dynamic and **e** static substrate. Fluorescent images of hiPSC-CMs staining with MYOM1and F-actin on **d** dynamic and **f** static substrate (scale bar: 10 μm)

BAG3 mutations are highly associated with DCM [31, 32]. WT and BAG3-/- iPSC-CMs were used to investigate myofibril remodeling under mechanical action. The iPSC-CMs were seeded on the Geltrex coated dynamic and static SMPs and cultured at 30 °C for 48 h to facilitate cell attachment. Next, the SMPs were activated at 37 °C to restore their original shape, a process that progressed rapidly within the first 2 h and was completed by approximately 5 h (Fig. 2a). This time point was selected as the critical window, as polymer shape recovery triggers mechanosensitive focal adhesion signaling that initiates myofibril remodeling of hiPSC-CMs [33]. Our prior work demonstrated that around 5 h post-activation, both Z-lines and M-lines reach peak density, followed by increased spacing, marking a transitional stage of myofibril remodeling [23].

We examined two conditions: static substrate (unstrained group) and dynamic substrate (40% strained group). The sarcomere structures were well-organized, composed of Z-lines, M-lines, and thin and thick filaments, enabling contractile activity (Fig. 2b). To analyze myofibril remodeling in hiPSC-CMs under dynamic (Fig. 2c, d) and static (Fig. 2e, f) conditions, we stained the sarcomere structures of individual hiPSC-CMs with α-actinin (Z-lines) (Fig. 2c, e) and myomesin (M-lines) (Fig. 2d, f). In addition, thin filaments stained by actin are anchored to the Z-lines and play a crucial role in contractile activity. Non-muscle-specific myosin (NM-II), stained with MYH10, is an essential actin-binding protein that surrounds *α*-actinin to facilitate the formation of pre-myofibrils during myofibril remodeling. Fluorescent imaging revealed the arrangement of myofibrils in hiPSC-CMs on both static and dynamic surfaces 5 h after the shape change (Fig. 2c–f). Individual cell images were analyzed to measure the cell area, cell elongation, and sarcomere organization.

**Fig. 3:**
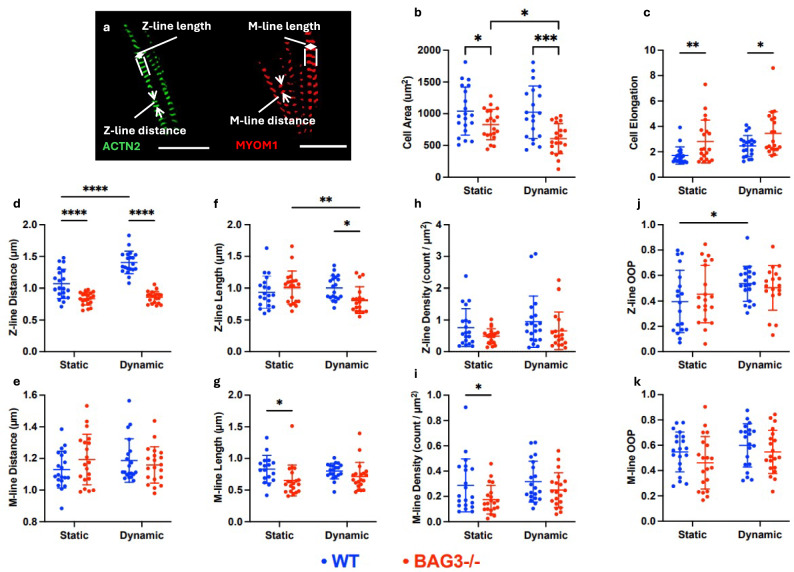
Myofibril remodeling of WT and BAG3-/- hiPSC-CMs was analyzed on both dynamic and static substrates at 5 h. **a** Fluorescent images were used to measure the lengths and distance of the sarcomere Z-lines and M-lines (Scale bar: 10 µm). **b** The cell area of BAG3-/- hiPSC-CMs was reduced on the dynamic substrate compared to the static substrate. **c** The elongation of WT hiPSC-CMs was less than that of BAG3-/- hiPSC-CMs on both substrates. WT and BAG3-/- hiPSC-CMs showed a significant difference in **d** Z-lines distances, **e** but not M-lines. **f** The Z-line length in BAG3-/- hiPSC-CMs decreased on the dynamic substrate. **g** M-line length showed a difference between WT and BAG3-/- hiPSC-CMs on static substrate. Sarcomere density only showed a difference on M-lines between WT and BAG3-/- hiPSC-CMs on static substrate. **j** Dynamic mechanical cues improved the Z-line alignment in WT hiPSC-CMs, but not seen from BAG3-/- hiPSC-CMs. **k** No difference was observed on M-line alignment. Statistics: two-way ANOVA with multiple *t*-test with *p*-value < 0.05 (*), < 0.01 (**), < 0.001 (***), and < 0.0001 (****), *n* ≥ 15 represents the number of cells collected from multiple SMP substrates

BAG3 plays a crucial role in autophagy and the maintenance of protein homeostasis. The relationship between myofibrillar integrity and BAG3 levels revealed Z-line degeneration under mechanical stress-induced apoptosis [34, 35]. Fluorescent images illustrate the Z-lines (green) and M-lines (red) of the sarcomeres (Fig. 3a). The cell area of BAG3-/- hiPSC-CMs was significantly smaller than that of WT in both static and dynamic substrates. Comparing the dynamic and static conditions, WT cells did not show significant differences in cell area, but BAG3-/- cells showed a significant reduction in cell area (Fig. 3b). The reduction in the area of BAG3-/- hiPSC-CMs indicated a failure to maintain proper cell skeletal structure under mechanical stress. Additionally, we calculated the cell aspect ratio (AR) as the cell elongation. BAG3-/- hiPSC-CMs exhibited higher elongation than WT hiPSC-CMs in both substrates after 5 h, indicating the thinning of cardiomyocyte shape seen in DCM due to dilation of the ventricular wall [36, 37] (Fig. 3c). For both cell types, there was no significant difference in the AR between the static and dynamic groups, indicating that cell elongation was not affected by dynamic mechanical changes at 5 h.

In dynamic mechanical environments, myofibrils undergo remodeling to adapt to the new mechanical environment [38]. BAG3 is associated with Z-lines in cardiomyocytes and plays a crucial role in stabilizing these structures [39]. In both the static and dynamic groups, WT hiPSC-CMs displayed significantly longer Z-line distances than BAG3-/- hiPSC-CMs (Fig. 3d). This suggests impairment of the Z-line structure in BAG3-/- hiPSC-CMs. The Z-line distance in WT hiPSC-CMs on the dynamic substrate was significantly increased compared to that on the static substrate, indicating that mechanical actuation promoted the sarcomere growth of WT cells [23]. No preferential changes in Z-line distance were observed in BAG3-/- CMs on either substrate, indicating that BAG3-/- CMs could not achieve such sarcomere growth similar to WT CMs. We also analyzed the M-line distance but found no differences between WT and BAG3-/- CMs on both substrates (Fig. 3e).

On the dynamic substrate, the Z-line length of BAG3-/- CMs was significantly shorter than that of the static substrate. More importantly, the Z-line length of WT CMs was significantly greater than that of BAG3-/- CMs on a dynamic substrate. The reduced Z-line length of dynamic BAG3-/- CMs evidenced Z-line impairment compared to WT CMs (Fig. 3f). Our work highlighted that dynamic mechanical actuation facilitates the identification of sarcomere disorganization, as evidenced by the Z-line distance and length. Additionally, the M-line length of WT hiPSC-CMs was slightly longer than that of BAG3-/- CMs under static conditions (Fig. 3g). No significant changes in the density of Z-lines or M-lines were observed under either condition (Fig. 3h–i). Sarcomere OOP was used to assess sarcomere alignment. WT CMs exhibited improved Z-line alignment on dynamic substrates compared with static substrates (Fig. 3j). However, no improvement in the Z-line alignment was observed in BAG3-/- CMs. In addition, there was no remarkable improvement in the M-line alignment in either WT or BAG3-/- CMs under dynamic conditions (Fig. 3k). M-lines are located in the middle of the sarcomere, connecting thick filaments, mainly stabilizing thick filaments, and are not involved in the contraction activity [40, 41]. Our results indicated that M-line structures were less sensitive to mechanical stress at 5 h, highlighted a temporal distinction in the assembly and regulation of Z-lines over M-lines.

### Sarcomere Structure and Contractile Function at 24 Hours After Shape Recovery Triggering

**Fig. 4:**
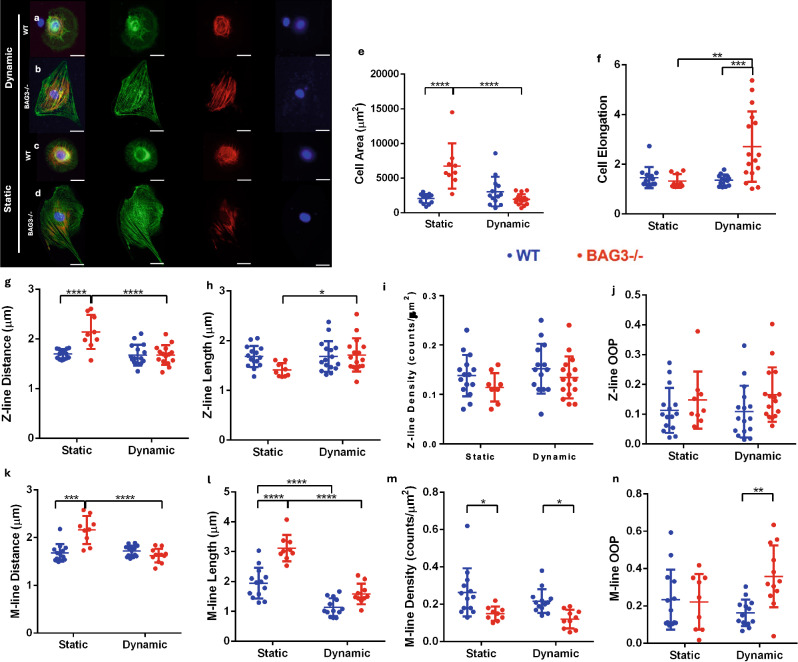
Myofibril remodeling in WT and BAG3-/- hiPSC-CMs on static and dynamic substrates after 24 h. Fluorescence images of WT (**a**, **c**) and BAG3-/- hiPSC-CMs (**b**, **d**) on static and dynamic substrates 24 h after shape, stained for Z-lines (ACTN2) and M-lines (MYOM1) (Scale bar: 10 μm) **e** BAG3-/- CM area on static substrates was significantly larger than both static WT CMs and dynamic BAG3-/- CMs. **f** BAG3-/- CMs on dynamic substrates were significantly more elongated compared with WT CMs on dynamic substrates and BAG3-/- CMs on static substrates. **g** Z-line and **k** M-line distance were greater in static BAG3-/- CMs compared with static WT CMs and dynamic BAG3-/- CMs. **h** Z-line length in static BAG3-/- CMs on was slightly greater than dynamic BAG3-/- CMs. **j** WT CMs displayed longer M-line length than BAG3-/- CMs on static substrates. Both WT and BAG3-/- CMs exhibited longer M-lines on static compared with dynamic substrates. **i** Z-line density and **j** Z-line orientation order parameter (OOP) showed no significant differences between WT and BAG3-/- hiPSC-CMs. **m** WT CMs exhibited higher M-line density than BAG3-/- CMs on both substrates. **n** M-line alignment was higher in BAG3-/- hiPSC-CMs cultured on dynamic substrates at 24 h Statistics: two-way ANOVA with multiple t-test with *p*-value < 0.05 (*), < 0.01 (**), < 0.001 (***), and < 0.0001 (****), n ≥ 12 represents the number of cells collected from multiple SMP substrates

To evaluate the long-term effects of substrate dynamics on myofibril remodeling, we analyzed WT and BAG3-/- iPSC-CMs on dynamic (Fig. 4a, b) and static (Fig. 4c, d) substrates at 24 hours after shape recovery triggering. Dynamic BAG3-/- CMs decreased cell area (Fig. 4e) and increased cell aspect ratio (Fig. 4f) compared to static BAG3-/- CMs, indicating an eccentric cell thinning phenotype commonly observed in myocardial dilatation. Interestingly, dynamic BAG3-/- CMs exhibited reduced Z-line distance (Fig. 4g) but increased Z-line length (Fig. 4h) relative to static BAG3-/- CMs. These changes were not observed between dynamic and static WT CMs, suggesting impaired Z-line remodeling and disrupted sarcomere growth in BAG3-/- CMs under dynamic mechanical actuation. However, we did not observe significant difference in Z-line density (Fig. 4i) or OOP (Fig. 4j) between WT and BAG3-/- CMs or between static and dynamic substrates. The most pronounced differences between short-term (5 h) and long-term (24 h) myofibril remodeling were observed in M-line measurements. While no significant changes were detected at 5 h, BAG3-/- CMs exhibited similar impairments in M-line organization as seen with Z-lines, including altered M-line distance (Fig. 4k) and M-line length (Fig. 4l) between static and dynamic substrates at 24 h. Notably, M-line density was significantly higher in WT CMs compared with BAG3-/- CMs across both substrates (Fig. 4m), whereas M-line OOP was slightly lower in WT CMs relative to BAG3-/- CMs under dynamic conditions (Fig. 4n). Together, these findings suggest that M-lines in BAG3-/- CMs display heightened sensitivity to dynamic mechanical actuation, but this phenotype emerges only after prolonged culture.

**Fig. 5:**
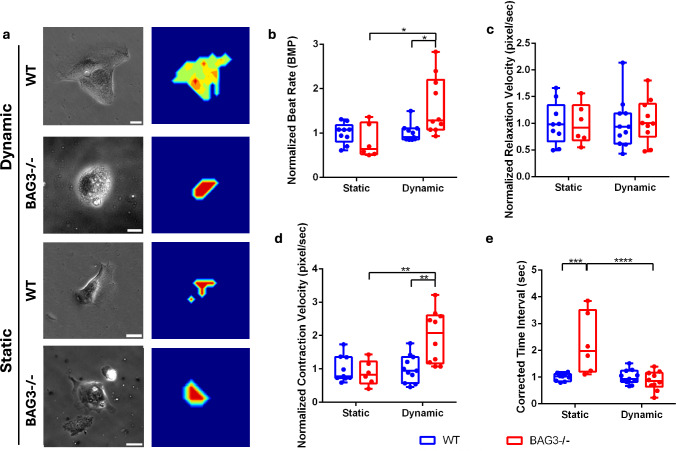
Contractile function of single hiPSC-CMs on static and dynamic substrates after 24 h. Beating activity was visualized using **a** motion velocity heatmaps (Scale bar: 25 µm). BAG3-/- CMs on dynamic substrates exhibited significantly higher **b** beat rate and **d** contraction velocity compared with WT CMs on dynamic substrates and BAG3-/- CMs on static substrates. WT CMs on static substrates demonstrated no significance on **c** relaxation velocity relative to dynamic substrates. BAG3-/- iPSC-CMs on static substrates showed longer **e** corrected contraction–relaxation (C–R) time interval compared with BAG3-/- CMs on dynamic substrates and WT CMs on static substrates. Statistics: two-way ANOVA with multiple *t*-test with *p*-value < 0.05 (*), < 0.01 (**), < 0.001 (***), and < 0.0001 (****), *n* ≥ 6 represents the number of cells collected from multiple SMP substrates

To further assess the functional behaviors of CMs under mechanical stress, single iPSC-CMs were recorded at 24 hours to generate beating behaviors of motion velocity (Fig. 5a). BAG3-/- iPSC-CMs on dynamic substrates exhibited a significantly higher beat rate (Fig.5b) and contraction velocity (Fig. 5d) compared with both WT iPSC-CMs on dynamic substrates and BAG3-/- iPSC-CMs on static substrates. The increase in contractile performance of BAG3-/- iPSC-CMs on dynamic substrates indicated a hypercontractile phenotype of BAG3-/- iPSC-CMs, as an early sign of cardiomyopathy. Relaxation velocity showed no significant differences between WT and BAG3-/- hiPSC-CMs or between static and dynamic substrates (Fig. 5c). In addition, BAG3-/- iPSC-CMs on dynamic substrates displayed a shortened contraction–relaxation (C–R) interval compared with BAG3-/- iPSC-CMs on static substrates, highlighting the sensitivity of BAG3 deficiency to mechanical stress (Fig. 5e).

## Discussion and Conclusion

In summary, this study underscores the profound influence of the dynamic extracellular mechanical environment on cardiomyocyte disease, particularly in the context of myofibril remodeling in both healthy WT and diseased BAG3-/- hiPSC-CMs. Using our SMP-based active biomaterial platform, we successfully induced dynamic mechanobiological conditions that drive myofibril remodeling in hiPSC-CMs. Under dynamic stress, BAG3-/- hiPS-CMs exhibited persistent lesions in sarcomere Z-lines, characterized by fractures and misalignment. BAG3 plays a pivotal role in maintaining the stability of cardiac sarcomeres by binding to actin-capping proteins. Mutations in BAG3 disrupt this function, leading to protein degradation within the Z-lines, ultimately resulting in myofibril disintegration. Our findings highlight the critical role of BAG3 in preserving the Z-line integrity during myofibril remodeling induced by dynamic mechanical stress. In our system, WT cardiomyocytes beat at an average rate of ~ 66 beats per minute (~1 beat per second), whereas the SMP shape recovery occurs gradually over several hours (~5 h to complete). Given this large difference in time scales—seconds for contraction–relaxation cycles versus hours for substrate deformation—the shape recovery provides only a very slow, quasi-static change in mechanical loading relative to the rapid contractile activity of the cells. Consequently, this slow deformation has minimal direct impact on the beat rate or contractile rhythm of WT CMs. However, at 24 h, BAG3-/- CMs exhibited a higher beat rate, likely reflecting their altered mechanosensitivity to dynamic stress over prolonged exposure. This study highlights the interplay between genetic deficiency and mechanical stress to facilitate disease progression in BAG3-associated DCM. Future investigations should focus on understanding how myofibril remodeling alters the contractile behaviors of WT and BAG3-/- hiPSC-CMs, providing deeper insights into the mechanobiological modeling of DCM under dynamic mechanical conditions.

## Supplementary Information

Below is the link to the electronic supplementary material.

Supplementary file1 (DOCX 831 KB)

Supplementary file2 (MP4 90 KB)

Supplementary file3 (MP4 44 KB)

Supplementary file4 (MP4 389 KB)

Supplementary file5 (MP4 217 KB)

## Data Availability

The raw and processed data required to reproduce these findings will be made available via contact with the corresponding author, Z.M. (zma112@syr.edu).
