# Supplementary material for: Disrupted Sarcomere Reorganization of Cardiomyopathy-Prone Human iPSC-Derived Cardiomyocytes on a Dynamic Mechanical Substrate
Source: Cell Mol Bioeng. 2025 Nov 21;18(6):661–72. doi: 10.1007/s12195-025-00880-z (PMC12664868; doi:10.1007/s12195-025-00880-z)
Supplement: Supplementary file 1 — Supplementary file1 (DOCX 831 KB) [file 12195_2025_880_MOESM1_ESM.docx]

**SUPPLEMENTARY INFORMATION**

**Disrupted Sarcomere Reorganization of Cardiomyopathy-Prone Human iPSC-Derived Cardiomyocytes on a Dynamic Mechanical Substrate**

Nhu Y. Mai^1,2^*, Xiangjun Wu^1,2^*, Huiyao Liu^1,2^, Ariel Ash-Shakoor^1,2^, Huaiyu Shi^1,2^, Zhuocheng

Qu^3^, Patrick T. Mather^4^, Xinrui Wang^3^, James H. Henderson^1,2^, Zhen Ma^1,2 #^

^1^Department of Biomedical & Chemical Engineering, Syracuse University, Syracuse NY, USA

^2^BioInspired Institute for Materials and Living Systems, Syracuse University, Syracuse NY, USA

^3^Department of Pharmacology, SUNY Upstate Medical University, Syracuse, NY, USA

^4^Department of Chemical Engineering, Penn State University, University Park, PA, USA

^#^Corresponding Author: Zhen Ma ([zma112@syr.edu](mailto:zma112@syr.edu))

Table S1. Antibodies used in this study.

| **Antibody** | **Cat.** | **Vendor** | **Dilution** |
| --- | --- | --- | --- |
| ACTN2 | A7811 | Sigma-Alrich | 1:200 |
| ACTN2 | 14221-1-AP | Proteintech | 1:200 |
| Myomesin | mMac myomesin B4 | DSHB | 1:13 |
| 488 goat anti-rabbit | A11008 | ThermoFisher | 1:200 |
| 488 goat anti-mouse | A-11029 | ThermoFisher | 1:200 |
| 546 goat anti-mouse | A11003 | ThermoFisher | 1:200 |


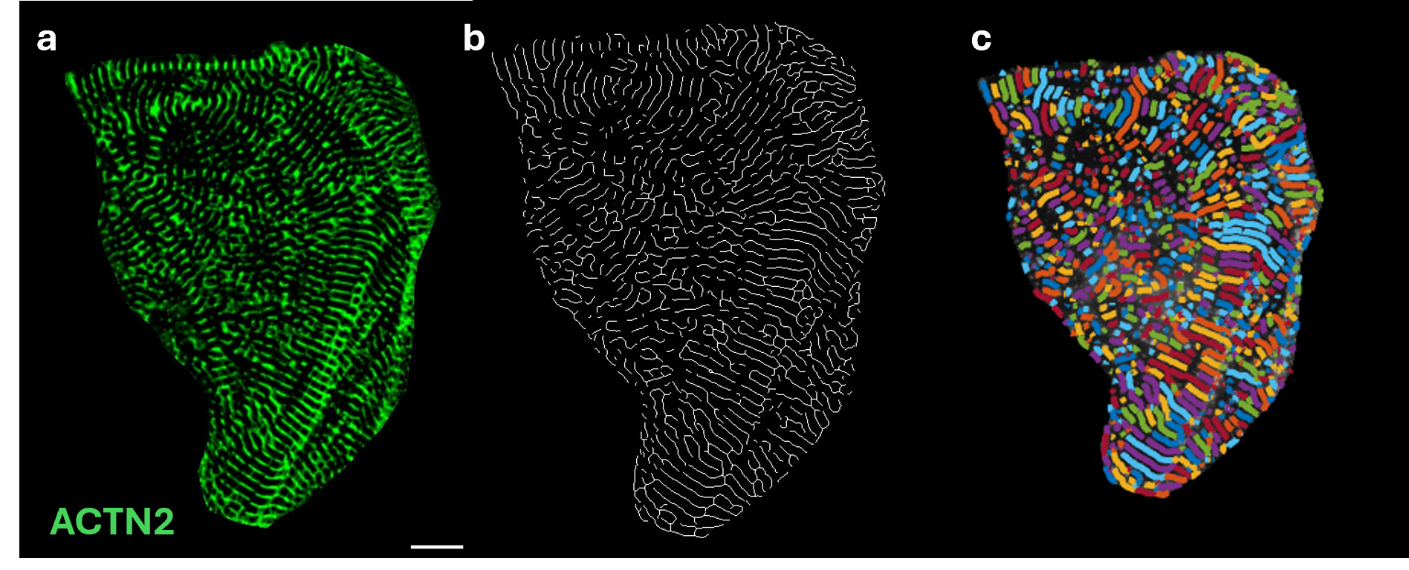


**Figure S1:** **Sarcomere images were analyzed using MATLAB algorithm.** (a) Fluorescent image of WT iPSC CM was used to convert into binary skeletons of myofibril images were analyzed to assess Z-line orientational order, and (c) color-coded segments indicate orientation and length, with measurements reported in pixels. (Scale bar: 10µm)

**Movie S1.** Video of beating WT hiPSC-CM on a static substrate.

**Movie S2.** Video of beating WT hiPSC-CM on a dynamic substrate.

**Movie S3.** Video of beating BAG3-/- hiPSC-CM on a static substrate.

**Movie S4.** Video of beating BAG3-/- hiPSC-CM on a dynamic substrate.
